# In This Issue

**Published:** 2001

## CIRCADIAN RHYTHMS

Throughout nature, the rhythms that govern everyday changes in behavior and physiology arise from a complex timekeeping system within the body. This timekeeping system, or biological clock, allows an organism to anticipate and prepare for changes in the physical environment that are associated with night and day, thereby ensuring that the organism does the “right thing” at the right time during a 24-hour cycle. The biological clock also provides internal temporal organization and ensures that internal changes occur in coordination with one another. The synchronization of an organism with both its external and internal environments is critical to its well-being and survival. Structurally, the biological clock governing these cycles is located in a brain region called the suprachiasmatic nuclei. Although the exact mechanisms underlying the body’s timekeeping system remain unclear, certain genes appear to play a prominent role. As reported by Drs. Martha Hotz Vitaterna, Joseph S. Takahashi, and Fred W. Turek, disruptions of normal circadian rhythmicity, such as that caused by alcohol consumption, have been associated with numerous mental and physical disorders and can impair safety, performance, and productivity. (pp. 85–93)

## ALCOHOL, CIRCADIAN RHYTHMS, AND TEMPERATURE

The complex interaction between alcohol and the body’s circadian rhythm has spawned a new research focus called chronopharmacology. This area has key implications for the field of alcohol research, because understanding alcohol’s effects on the body’s internal clock will aid scientists in designing medications and devising behavioral interventions for treating alcohol abuse and dependence. Drs. Jill A. Wasielewski and Frank A. Holloway review studies examining how alcohol and the body’s circadian rhythm interact using body temperature as an index of circadian-rhythm function. Though this research is not extensive, findings indicate that alcohol sensitivity and preference for drinking do indeed appear to vary with circadian timing and that alcohol may be acting directly on the central pacemaker to alter circadian functioning. (pp. 94–100)

## SLEEP, SLEEPINESS, AND ALCOHOL USE

In healthy, nonalcoholic people, moderate alcohol consumption initially may improve sleep by decreasing the length of time needed to fall asleep. Increased alcohol consumption, however, can disrupt sleep by disturbing the critical second half of the sleep period and by altering the proportions of important sleep stages. Such sleep disturbance may be mediated by alcohol’s effects on the hormones and brain chemicals involved in sleep regulation. Drs. Timothy Roehrs and Thomas Roth explore the relationship among alcohol-related sleep disruptions, daytime sleepiness, and alcohol-induced performance impairments. For example, the level of sleepiness or alertness at the time of alcohol consumption influences alcohol’s subsequent sedating and performance-disrupting effects. Drs. Roehrs and Roth also discuss the hypothesis that variations in both the duration of nighttime sleep and the level of daytime sleepiness may, in turn, play a role in modulating alcohol consumption. (pp. 101–109)

## ALCOHOL’S EFFECTS ON SLEEP IN ALCOHOLICS

Compared with nonalcoholics, alcoholics experience a greater number of sleep problems, such as difficulty falling asleep and a decrease in total sleep time, reports Dr. Kirk J. Brower. Other measures of a good night’s sleep, such as the percentage of sleep time spent in deep sleep (i.e., slow-wave sleep) or rapid eye movement (REM) sleep, also are altered. These problems occur not only during periods of heavy drinking, but also during alcohol withdrawal and following short or prolonged periods of abstinence. Such persistent sleep problems, even during prolonged abstinence, may increase the risk of relapse to drinking for some alcoholics. By addressing sleep problems during alcoholism treatment, it may be possible to reduce the rate of relapse. Sleep problems also may predispose some people to alcoholism: Up to one-fourth of people with insomnia report having used alcohol to self-medicate their problems. (pp. 110–125)

## ALCOHOL, ANTIDEPRESSANT, AND CIRCADIAN RHYTHMS

Alcohol consumption (both acute and chronic) and alcohol withdrawal have a variety of chronobiological effects in humans and other animals. Those effects are widespread, altering the circadian rhythms of numerous physiological, hormonal, and behavioral functions. Dr. Alan M. Rosenwasser focuses on the neurochemical effects of alcohol within the body’s timekeeping circuitry. Those studies suggest that the effects of alcohol on animal circadian rhythms are similar to those seen when administering antidepressant drugs, and alcohol’s effects on human circadian rhythms during withdrawal are reminiscent of those described in depressed patients. These observations suggest that alcohol may produce antidepressantlike effects on the circadian pacemaker. One theory posits that the effects of alcohol on the circadian pacemaker are mediated in part by alterations in a key chemical involved in cellular communication (i.e., serotonin) within the circadian system. (pp. 126–135)

## DEVELOPMENTAL ALCOHOL AND CIRCADIAN CLOCK FUNCTION

A wide variety of physiological processes and body functions are regulated by the body’s internal clock, which is located in the suprachiasmatic nuclei (SCN) in the brain. Drs. David J. Earnest, Wei-Jung A. Chen, and James R. West discuss the hypothesis that exposure to alcohol during pregnancy may result in permanent damage to the internal clock of the fetus and that such damage may contribute to the behavioral impairments and affective disorders commonly found in people with prenatal alcohol exposure. To date, this hypothesis has been explored primarily in animal models. According to preliminary findings, adult rats exposed to alcohol during a critical period of brain development experience a shortened circadian sleep-wake cycle than unexposed rats, suggesting an alcohol-induced disturbance of the internal clock. The exposed animals’ activity also was more fragmented, with frequent shifts between shortened periods of sleeping and wakening. (pp. 136–140)

## CHRONOBIOLOGICAL REGULATION OF ALCOHOL INTAKE

Acohol consumption, like food intake in general, appears to be influenced by circadian rhythms. For example, in rodents, which are most active at night, both food and fluid intake—including alcohol intake—generally occur during the animals’ active dark phase. Drs. Susanne Hiller-Sturmhöfel and Paul Kulkosky review some of the mechanisms and factors that may regulate or modulate circadian consumption patterns. For example, the hormone melatonin, which is produced in the pineal gland, may help control circadian alcohol-consumption patterns. Other factors— such as the specific animal model being studied, the alcohol administration schedule, or disruptions of the normal daily cycle—also can affect circadian regulation of alcohol intake. (pp. 141–148)

